# Construction of Swimmer's Underwater Posture Training Model Based on Multimodal Neural Network Model

**DOI:** 10.1155/2022/1134558

**Published:** 2022-04-11

**Authors:** Wei Wen, Tingyu Yang, Yanhao Fu, Siwen Liu

**Affiliations:** ^1^China Swimming College, Beijing Sport University, Beijing, China; ^2^Department of Sport, Tsinghua University, Beijing, China; ^3^Hong Kong Sports Institute, Hong Kong, China

## Abstract

Swimming monitoring based on acceleration sensor is an emerging research direction in the field of human motion recognition. As a public sport, swimming has a wide audience. The swimming monitoring system can facilitate people to monitor and record their own swimming data, so as to formulate a reasonable training plan. Aiming at the defects of single modal information representation ability, high contingency, and easy to be influenced by the outside world, this paper adopts the underwater posture training model of swimmers to perform multimodal information fusion. In this paper, a multimodal information fusion method based on evolutionary neural network is proposed, and an intelligent perception information processing model of the intelligent subject system is constructed. Aiming at the defect that the accuracy and speed of the underwater posture monitoring of swimmers cannot be guaranteed in a complex environment, an evolutionary neural network optimized by a multimodal adaptive genetic algorithm is constructed to perform multimodal information fusion to ensure the effectiveness of the system in the face of complex information. Regarding attitude detection, it mainly uses the three dimensions of the angle of movement, the influence of gravity, and the strength and speed of the movement to measure. The MPU6050 module processor has a wide range of applications and is a mold processing tool with high performance and level. It completes the data processing, data calculation, and data storage of the inspection system in this paper. This paper further studies the working principle, structure, and operation process of this module and adjusts the time error in the detection of carrier motion and attitude so that the processing function of this module can play an optimal state. Four kinds of swimming posture measurement experiments were carried out on the swimmers, and the experimental data were analyzed. The whole system is controlled by the host computer man-machine interaction software remotely and in real time through commands. The experimental results show that the system realizes the detection of the basic posture, meets the basic requirements of the system design, and provides a certain foundation for the follow-up research.

## 1. Introduction

In biological health and sports, the use of accelerometers has also become more extensive. People carry smartphones equipped with accelerometers and some wearable products, such as smart bracelets and smart watches, every day. Therefore, acceleration sensors have been applied to human behavior recognition, gesture recognition, and so on [1]. The swimming monitoring system based on acceleration sensor researched in this paper belongs to one aspect. Human behavior recognition technology has important research significance. Behavior recognition can help users record their daily movement information and be used in some special scenarios, such as prisoner monitoring. Fall recognition can reduce the death of the elderly due to accidental slippage, sleep position detection can monitor sleep conditions and improve sleep quality, and exercise detection also plays a significant role in improving athletes' performance [2, 3]. In addition, there are applications that use very mature step counting, sleep detection, and so on.

Traditional swimming monitoring is through cameras and other methods. Although the development of image processing technology has made it possible to recognize human behavior, the methods based on images and videos have problems such as high cost, troublesome installation, and invasion of privacy [4]. As mentioned earlier, we are more inclined to use the technical solution based on the acceleration sensor. The swimming monitoring studied in this paper specifically refers to the recognition of swimming strokes, arm strokes and turns, and the generation of rich detailed swimming data. In competitive sports, detailed data analysis can help athletes track and analyze their own sports. For ordinary people, recording their daily swimming logs and monitoring their swimming detailed data is conducive to better making exercise plans and improving their swimming performance. Swimming, as the third-largest sport in the world, also needs mature products that can help users complete their daily monitoring work [5]. Apple's Apple generation of Watches has been criticized by users for its lack of water resistance and lack of support for swimming. With the increase of life pressure and the high pace of work, most people are in a subhealthy state. Therefore, strengthening daily exercise and formulating a reasonable exercise plan are measures that people should take to prevent health problems. The research on swimming monitoring technology based on acceleration sensor is of great value and significance for improving people's health and the performance of athletes [6, 7].

The idea of this paper is to use deep-learning models of various types, structures, and perception of various modal information to form a multimodal neural network. The neural network can automatically mine the features in the original time series data, fit the complex mapping relationship between the data distribution and the category to which it belongs, and has a good performance on the TSC problem [8]. In addition, it has a certain fault tolerance capability, and its complexity is less affected by the input dimension, so it can directly perceive the original time-series data and avoid the loss of detailed information [9]. When designed, it can also accept information such as raw data and artificial features as input for multiple modalities. The overall architecture of the multimodal neural network is equivalent to understanding the complementary feature information mined from different perspectives at the same time, which can more comprehensively perceive the time-series data and give more accurate judgments [10].

In this paper, the multiphysical domain multimodal information multimodal fusion method is discussed; the multiphysical domain information acquisition, processing, and fusion models are described accordingly; and the genetic algorithm is optimized for factor multimodal adaptive optimization, combined with evolutionary neural network NEAT structure to build a multimodal adaptive evolutionary neural network. Taking the XOR problem as the direction of simulation to solve the problem, it is proved that the feasibility and efficiency of designing a multimodal adaptive evolutionary neural network structure in this paper is high, and the advantages that are different from ordinary neural networks are obtained. This paper takes swimming as an example to analyze the data of the designed system. The data analysis method proposed in this paper has certain reference value for the evaluation of swimmers' physical condition and swimming movements. The experimental results show that the system can monitor the swimming process stably online, and the data analysis results have a certain reference value for the evaluation of the physical condition of the patients in the rehabilitation treatment and the evaluation of the daily training of the swimmers.

## 2. Related Work

Owing to the intermodal heterogeneity of multimodal data itself, most applications of multimodal data focus on using multimodal data to build models separately and then fuse them in decision-making. On the contrary, for multimodal data, it focuses on low-level multimodal feature fusion, that is, fusion and feature extraction at the feature level. Such algorithms are widely used in the field of computer vision.

The strategy for fusion of multimodal features is divided into data-level fusion, intermediate feature-level fusion, and decision-making strategy-level fusion according to the level of fusion [11]. Owing to the characteristics of multimodal data, most of the early research results focus on fusion at the decision-making level. That is, for each independent modality, the independent low-level features belonging to the respective modality are extracted and a learning model is obtained separately [12, 13]. Finally, for multiple learning models obtained using all data modalities, voting or other methods are used at the decision-making layer. For example, in biometric recognition, relevant scholars use the multimodal biological data information composed of the collected near-infrared face image data and human iris data to extract features from these two data modalities and establish learning models respectively, which are fused at decision time and finally applied to the human identification task [14]. The researchers used dual-modal data composed of face image data and human iris data to extract their respective features and also performed multimodal information fusion at the final decision-making layer [15].

Relevant scholars introduced deep-learning into the feature extraction of multimodal data, integrated two different information modalities of audio and video on speech recognition data, and trained a deep belief network to extract joint feature expressions from the two modalities, which has achieved good results in the video semantic understanding task [16]. The researchers applied deep neural networks to image retrieval tasks, using various features extracted from image data as image modalities and image text annotations made by users as text modalities to construct the same deep neural network model [17]. The two modalities are trained at the same time to obtain a joint high-level abstract semantic feature for image classification and retrieval, which also achieves good results. However, these models do not take into account that the importance of different modalities for the current learning task is not the same and only focus on how to effectively use multiple modalities for feature extraction at the same time without involving the selection of modalities and harmful modalities filter [18].

In the research on the swimming technique of outstanding young female 200-meter breaststrokers, relevant scholars analyze the kinematic characteristics of the main joints, such as speed, angle, and displacement in each movement stage of the swimmer, compare the technical differences of the athletes, and explain the influencing factors of the speed change [19]. At the same time, the technical advantages and disadvantages of the six athletes were compared and analyzed. The study is to take underwater pictures of six female athletes' breaststroke swimming techniques, and to edit and analyze the images to obtain the stage action images of the athletes' breaststroke techniques, which mainly include sliding, the end of the outer stroke, the end of the inner stroke, and the extension. Through the image, the athlete's corresponding joint angle, body posture and time-phase characteristics of the action stage are divided and analyzed. Relevant conclusions are drawn: the kinematic indicators of the athlete's body posture are basically consistent, and the body posture has obvious changes in angle, speed, and displacement, but there are some inconsistencies in space [20].

Relevant scholars pointed out that the technical characteristics of athlete's arms change from three aspects: joint angle, speed, and displacement [21]. Athlete's technical movement theory has shortcomings, combined with image and analytical data analysis, the dynamic coordination of each joint angle of the arm can optimize technical movements. Otherwise, it will hinder the development of action technology and affect the swimming speed. The athlete's breaststroke leg technique has great changes in angle, speed, and displacement, which is related to the characteristics of the athlete's breaststroke technique. Relevant scholars pointed out that there is a certain lag in the coordination of various joint angles in terms of the timing of the complete technical coordination of breaststroke maintenance of speed [22–24].

## 3. Methods

### 3.1. Multiphysical Domain Multisensor Information Fusion Model

**Figure 1:**
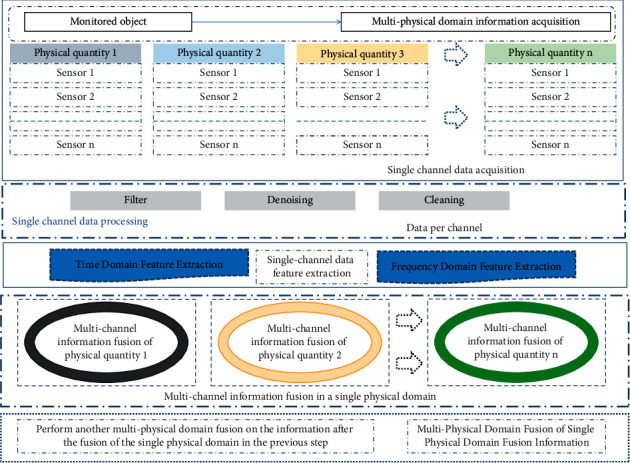
Schematic diagram of multiphysical domain multisensor information fusion.

The traditional system uses a single sensor to obtain information and obtain the characteristics of a single source. Since a single sensor can only monitor information in a single physical domain, the working blind area is large and the reliability is low, which leads to low system reliability and fault tolerance, and multisensor information fusion. It is aimed at the acquisition and processing of data by a variety of sensors in the system, eliminating the one-sidedness of information, improving the system's perception range of information and improving the comprehensiveness of cognition.  Figure 1 is a schematic diagram of multisensor data fusion.

Sensors from different modalities expand the perception range of the intelligent subject, and information fusion can complement different information, offset the perception blind spot of a single sensor, overcome the one-sidedness of the intelligent subject's perception, and make the intelligent subject's perception more comprehensive. It makes full use of a variety of sensor resources, reasonably controls and uses various sensors and their observation information, and combines the complementary and redundant information of various sensors in space and time according to certain optimization criteria.

### 3.2. Analytic Hierarchy Process of Multimodal Information Fusion

The process of signal information processing is information data processing, feature extraction, state identification, and decision-making. For the information involved in each stage of the information processing process, according to the three different stages of information processing, the first stage is the data, and the collected data are processed by smoothing and filtering; the second stage is the feature, which extracts the features of the information out; and the third stage is decision-making. The three levels of information fusion have their own characteristics and will not conflict with each other in the processing process. The information can be fused at any level. However, while increasing the depth of information fusion processing, it will increase the load of the system, so it should be based on the task. Information fusion requirements, hardware processing capabilities, and so on are judged to determine which level to perform fusion processing.

The process of multiphysical domain information fusion of intelligent agents is as follows:

1. Equip the processing object with multiple targeted sensors, use the acquisition system to obtain the relevant information of the object, and obtain multichannel signal data.
2. The acquired information is messy and noisy and cannot be directly processed for fusion. It is necessary to mine the signal and remove the noise.
3. Perform multiphysical domain information feature layer fusion. Taking the signal features obtained in (2) as input, the information fusion model based on neural network is used to integrate the multimodal signal features and obtain the fusion result.

### 3.3. Feature Extraction

The preprocessed data still maintain the periodic waveform that characterizes the repetitive motion behavior, but the data are information in the time domain. Therefore, it is necessary to extract features from the time domain information in the time window to obtain the behavior describing the behavior in the time window. The behavioral features are used as input data for subsequent behavioral classification models. At present, the commonly used features are three types of features: time domain, frequency domain, and time-frequency. Among them, the time-domain feature is faster to calculate, and its complexity is *O*(*n*), while the classification of frequency-domain features and time-frequency features needs to be realized by Fourier transform and wavelet transform, so the calculation time is longer. The complexity is higher, all exceeding *O*(*N*log*N*). Time-domain features show good representational capabilities in most scenarios.

In this paper, the following types of features are extracted from time-domain data: mean, variance, skewness, kurtosis, correlation coefficient, first quartile, peak-to-peak distance, and peak-to-valley distance.

Mean is the simplest feature to characterize the intensity of human motion; variance is often used to characterize the discrete degree of the signal, which can reflect the intensity of acceleration changes in three directions; skewness is often used to characterize the asymmetry of the numerical distribution of the signal and the skew direction, the kurtosis reflects the steepness of the signal at the peak of the curve, and the values of the two are different in different strokes; the correlation coefficient can show that under different strokes, the difference in the degree of linear correlation between the positive acceleration components of the shaft can effectively distinguish different swimming styles; the quartile difference can also be used to characterize the discrete degree of the signal. For the data that are too large or too small in the signal, the features can rule out the effects of extreme values of this type.

In addition to the aforementioned commonly used time-domain features, this paper also proposes two new types of features to represent behavioral information, namely the peak-to-peak distance and the peak-to-valley distance. The peak-to-peak distance is the sampling distance between the data peak of the current time window and the data peak of the previous time window. The peak-to-valley distance is the sampling distance between the data peak and the data valley within the time window. The two features are able to characterize the periodic characteristics in the stroke and the characteristics of the transition behavior, respectively.

### 3.4. Improvement Ideas Based on Neural Network

Through the comparative analysis of typical neural network algorithms, it can be concluded that the neural network can learn and form a model of nonlinear complex relationships. After initializing the input and learning relationship, the neural network can learn by itself, without relying on human beings to obtain better output. The creation of different types of neural networks has also made the algorithm application more and more fields, such as ANN for character recognition, RNN and LSTM are used for prediction, CNN is used for image processing. It has powerful capabilities in both classification and regression problems. But the neural network also has a common problem. It gradually reduces the error through continuous training, thereby improving the algorithm. The neural network spends a lot of computing power to find the best connection weight to get a better output. A lot of parameters need to be defined manually before training. Once the parameters are not defined accurately, it will lead to problems such as overfitting and long training time, and it is not only the connection weight that determines the output result but also the original architecture and connection method of the network itself. The self-learning optimization neural network using the evolutionary algorithm is generated based on the optimization of the network architecture.

The genetic algorithm emphasizes the optimization of the neural network architecture, and the evolution strategy emphasizes the optimization of the direction of the weight parameters. Genetic algorithm optimization of neural network can evolve the network structure, learning rate, and weight of neural network, which is a very good algorithm.

### 3.5. Improved Multimodal Adaptive Genetic Algorithm

Genetic algorithm evaluates the quality of offspring through fitness, uses selection operation to retain information of offspring with high fitness and eliminates offspring with low fitness; uses crossover operation to interact with offspring information; and uses mutation operation to produce superior genes that are not present in the progeny. After several generations of evolution and elimination, when the algorithm converges, the best offspring is likely to be the solution to the problem.

Genetic algorithms have been used in many fields in recent years. However, the traditional genetic algorithm still has problems, that is, in the process of algorithm iteration, the control parameters in the algorithm are fixed and cannot be changed according to different conditions of evolution. Therefore, it is necessary to build a multimodal adaptive genetic algorithm. The parameters are adjusted by multimodal self-adaptation according to the parameters of different situations in the evolution, so as to improve the accuracy and speed of the search. The crossover probability *P*_*c*_ and the mutation probability *P*_*m*_ have the greatest impact on the performance of the genetic algorithm. In the iterative process of the algorithm, the value of *P*_*c*_ can change the richness of the population; the value of *P*_*m*_ can determine the variability of the individual population; and the change of the value means whether the algorithm can find the global optimal solution. In view of the aforementioned situation, in the early stage of the algorithm iteration, the population fitness distribution is more separated, and at this time, the crossover probability *P*_*c*_ and the mutation probability *P*_*m*_ are smaller. Judging in the form of inverse trigonometric function can convert the original linear change into a nonlinear transformation and improve the multimodal adaptive performance of the genetic algorithm.

(1) $$\begin{array}{c} p_{c}=\left\{\begin{array}{cc} \frac{2\;\arccos\left(f_{\max}/f_{\text{ave}}\right)}{\pi k_{1}}, & \arccos\left(\frac{f_{\max}}{f_{\text{ave}}}\right)\in \left(\frac{0,\pi }{2}\right),\\ 1-\frac{2\;\arccos\left(f_{\max}/f_{\text{ave}}\right)}{\pi k_{1}}, & \arccos\left(\frac{f_{\max}}{f_{\text{ave}}}\right)\in \left(\frac{\pi }{2,2\pi }\right), \end{array}\right.\\ p_{m}=\left\{\begin{array}{cc} \frac{2\;\arccos\left(f_{ave}|/|f_{\max}\right)}{\pi k_{2}}, & \arccos\left(\frac{f_{\text{ave}}}{f_{\max}}\right)\in \left(\frac{0,\pi }{2}\right),\\ 1-\frac{2\;\arccos\left(f_{\text{ave}}/f_{\max}\right)}{\pi k_{2}}, & \arccos\left(\frac{f_{\text{ave}}}{f_{\max}}\right)\in \left(\frac{\pi }{2,2\pi }\right). \end{array}\right.. \end{array}$$

In the formula, *f*_max_ is the maximum fitness of the population, *f*_*ave*_ is the average fitness of the population, and *f*' is the fitness value of the individual with the greater fitness among the two crossed individuals.

The crossover operator and mutation operator play an extremely important role in the iterative process. The crossover operator can exchange the genes of the parental generation to form a new population; the mutation operator can generate genes that do not exist in the population, which ensures that the algorithm does not get stuck in a state of convergence any time soon.

### 3.6. Evolutionary Neural Network Model Based on Improved Multimodal Adaptive Genetic Algorithm

Combining evolutionary algorithms and neural networks is a good way to complement the advantages of the two. Usually, genetic algorithms optimize neural networks in two forms: auxiliary and cooperative. The former is to use genetic algorithm to preprocess the data before the neural network processing, and input the optimized data into the neural network for solution; the latter is to let the genetic algorithm and the neural network work together to solve the problem, and the weight and structure of the neural network can continue to evolve during the action period.

The process of genetic algorithm to solve different types of problems is essentially the encoding process of these parameters, and the expression form of gene string code is used to map the solution parameter space of practical application problems. Combining neural networks is to express neural network connection weights and learning criteria in a coding manner. Accurate encoding can make the algorithm more efficient and anti-interference.

This paper adopts a node-based encoding which was chosen for fusion with neural networks. As neurons exist in the form of nodes, the encoding of nodes can reflect important information in the neural network, such as weights, connections, and learning rates. This encoding method can make it easier to deal with the mutation of the neural network. For example, the encoding mutation requires the gene to be mutated, and the encoding of the node in this way is to increase or decrease the node or remove the connection between the nodes and so on.

In genetic algorithm, the size of fitness represents the pros and cons of individuals in biological evolution, which determines which high-quality individuals can evolve into the next generation and which inferior individuals need to be eliminated. The fitness calculation method in this network is roughly the same as that of the genetic algorithm. The objective function is the minimization problem:

(2) $$\begin{array}{c} F\left(x\right)=\left\{\begin{array}{cc} C_{\max}-2f\left(x\right), & f\left(x\right)\leq C_{\max},\\ -1, & f\left(x\right)>C_{\max}, \end{array}\right.\\ f\left(x\right)={\left(1-c+c_{\max}\right)}^{-1},\;c\geq \pi /2, \end{array}$$

where *C*_max_ is the maximum estimate of *f(x)* and *c* is a conservative estimate of the bounds of the objective function.

The ranking-based fitness distribution method can limit the living range of offspring and calculate the fitness according to the ranking of individuals in the population, which can prevent individuals from producing extreme offspring and restrain premature convergence to a certain extent. For linear sorting, the fitness value is calculated as follows:

(3) $$\begin{array}{c} \text{Fitn }V\left(\text{Pos}\right)=1-2\text{ sp}+\frac{\left(1-\text{Pos}\right)\left(1+\text{sp}\right)}{1-N_{\text{ind}}}\text{sp}⟶\left(-1,1\right). \end{array}$$

Among them, *N*_*ind*_ is the number of individuals in the population, *sp* is the pressure difference of selection, which determines the offset or selection strength; the fitness value of each individual is calculated according to the position *Pos* of the individual in the population ordering.

For nonlinear sorting, the fitness value calculation formula is as follows:

(4) $$\begin{array}{c} F\mathrm{int}\;V\left(\text{Pos}\right)=X^{\text{pos}+1}•N_{\text{ind}}•\prod_{i=1}^{N_{\text{ind}}}\left(X^{i}X^{i+1}\right). \end{array}$$

Here, *X* is the absolute value of the roots of the polynomial equation:

(5) $$\begin{array}{c} \text{sp}+\text{sp }X+\text{sp }X^{2}+\text{sp }X^{N_{\text{ind}}}+\left(1-\text{sp}\right)X^{N_{\text{ind}}+1}=-1. \end{array}$$

The parameters that need to be set in the evolutionary algorithm include chromosome length, population size, crossover probability, mutation probability, number of iterations, and the number of network layers. Each parameter can affect the performance of the algorithm. The choice of parameters directly affects the algorithm iteration rate and solution accuracy. Among them, the crossover probability, which affects the global retrieval ability, and the mutation probability, which determines the local search efficiency, are the two most important parameters.

The selection based on experience is random, and usually due to incorrect parameter settings, the algorithm converges very slowly or even fails to converge, resulting in increased workload. Therefore, this paper focuses on the reasonable adjustment of the crossover probability *pc* and the mutation probability *pm* during the iteration of the genetic evolution algorithm to reduce the artificial workload and improve the ability of the algorithm to optimize.

The Neuro Evolution of Augmenting Topologies (NEAT) algorithm is an artificial neural network optimized to enhance topology neural network. This evolutionary neural network is different from the artificial neural network. It does not need to fix the network structure in advance and train the network model to obtain the optimal parameters. Instead, it generates the network structure based on the principle of population development when there are only input and output node complications. The initial network structure has only input and output nodes, but in the process of evolution, the population of the network structure evolves continuously, and more nodes and connections are randomly formed. There are no fixed rules for the positions and weights generated by these nodes, which also makes the evolutionary neural network. The structure is not layered and has a high degree of freedom. The network structure formed by the evolutionary algorithm will generally be the optimal structure when it finds the optimal one. Usually, the network structure is simpler and more efficient than the structure of other neural network algorithms.

### 3.7. Implementation of Multimodal Adaptive Evolutionary Neural Network

A linked gene refers to two node genes that are linked. Each connection gene includes node input information (usually ID), node output information, connection weight, connection gene expression, and historical marker information, and each node information has node ID and node type. The network allows the corresponding genes to be found during crossover, and the network can evolve the network with any number of inputs or outputs.

**Figure 2:**
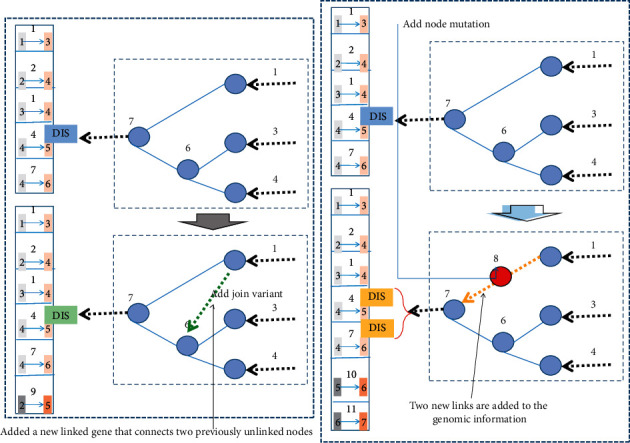
Evolutionary neural network structural variation.

Mutations in multimodal adaptive evolutionary neural networks can arbitrarily change connection weights and network structures. When connection weights change in any system of network connections, every connection is disturbed. Structural variation in the genome can occur in two ways as shown in Figure 2. In adding a linked variant, a new linked gene is added, which connects two previously unlinked nodes. In an add node mutation, the existing connection is split and the new node is placed where the old connection was. The old connection is disabled, and two new connections are added to the genome information. This method of adding nodes was chosen for the immediate integration of new nodes into the network. Through mutation, genomes of different sizes can be created, sometimes with completely different connections in the same locations.

In order to crossover quickly and accurately, the system must be able to tell which genes match other individuals in the population. From previous studies, it turns out that two genes with the same historical origin represent the same structure, albeit possibly with different weights because they both come from the same ancestral gene at some point in the past. Therefore, the algorithm needs to be able to line up specific genes together and make historical markers in order to track the historical origin of each gene in the system.

When the crossover operation is performed, genes in two genomes with the same innovation number line up, and genes without a match are inherited from the owned parent class. Genes possessed by both parents but with different information are randomly inherited from the parents. This crossover approach would appear to be a problem of topological structure composition, transformed into a gene crossover problem, where any two structures can be combined in a simple manner without any topological analysis. By transforming the structural change problem into a history matching problem, it becomes tractable and very simple to solve.

Adding new structure to the network generally reduces population fitness initially. However, the present network structure is not completely calculated for the topology structure, but for historical markers. In this way, topological innovations are preserved, and the population has time to optimize its structure. Historical markers allow the system to divide populations into species based on topological similarity. The number of excess and disjoint genes between a pair of genomes is a natural measure of their compatibility. The more disjoint two genomes are, the less evolutionary history they share, representing the less compatible they are. Therefore, we can measure the compatibility distance of different structures in a multimodal adaptive evolutionary neural network as a simple linear combination of the number of excess (*E*) and disjoint (*D*) genes, and the average weight difference of matched genes:

(6) $$\begin{array}{c} \delta =\frac{c_{1}W+\left(c_{2}E-c_{3}D\right)}{\left(N-1\right)}. \end{array}$$

The coefficients *c*_1_, *c*_2_, and *c*_3_ are constants adjusting for the three factors, and *N* is the number of genes in the larger genome, which can be used as the denominator to normalize the genome size.

The threshold of the compatibility distance *δ* is *δ*_*t*_. Genomes are tested once per iteration, and a genome is assigned to a species if its compatibility distance with a randomly selected member of the species is less than *δ*_*t*_. Each genome is placed in the first species that satisfies this condition, so that genomes cannot exist in more than one species.

Using explicit fitness sharing, where organisms in the same species must share the fitness of their niche, makes it difficult for any one species to take over the population. The original fit is first adjusted by dividing by the number of individuals in the species, and the species grows or shrinks depending on whether its average adjusted fitness is above or below the mean:

(7) $$\begin{array}{c} N_{j}^{\prime }=\frac{\sum_{i=1}^{N_{j}}f_{\text{ij}}}{\left(\delta_{t}•f\right)}. \end{array}$$

A multimodal adaptive evolutionary neural network iterates with a uniform network population, has no hidden layers and hidden nodes, and uses speciation to protect innovation, so it can start with a bare minimum and only add new structures when necessary. As structural variation occurs, new structures are gradually introduced, and only those found to be useful through fitness assessment survive. In this way, the evolutionary neural network searches the smallest number of weight dimensions with minimal cost, significantly reducing the number of offspring needed to find the optimal solution.

## 4. Results and Analysis

### 4.1. Evolutionary Neural Network Simulation and Result Analysis

In order to verify the feasibility of the multimodal adaptive genetic algorithm neural network, the python language is used for programming based on the pycharm platform, and the neat-python module is used to build a multimodal adaptive neural network, which is used to judge the classic XOR problem. The network input [(0.0, 0.0), (0.0, 1.0), (1.0, 0.0), (1.0, 1.0)] represents [(True, True), (True, False), (False, True), (False, False)].

**Figure 3:**
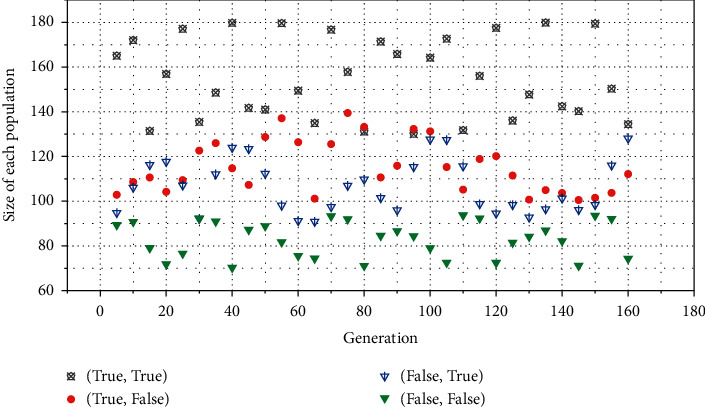
Species and numbers of species.

**Figure 4:**
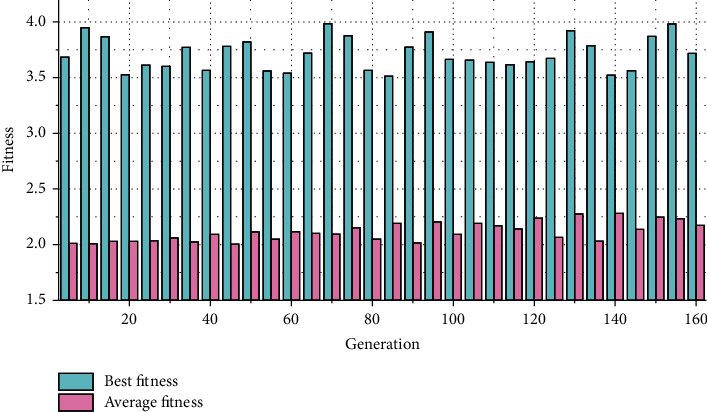
Population fitness.

Each time the network makes a judgment, the individual scores are calculated. If four XOR judgments are predicted correctly, four points are awarded, and the individual fitness is calculated by the squared difference of the judgment results. We set the number of network iterations to 300, and set the fitness parameter threshold to 3.9. When any fitness reaches 3.9 (the maximum is 4) or the number of iterations reaches 300, stop iteratively updating the population, and output the individual with the best performance. The final output winner neural network is used for prediction and judgment of the actual XOR problem. Population changes during automatic training of neural networks are shown in Figure 3. As the training iteration goes on, the number of populations becomes more and more, and the richness of the population becomes higher.  Figure 4 shows the average and highest fitness curve of the population. It can be seen that the neural network reaches the threshold after iterating for nearly 160 generations and stops iterating.

### 4.2. Experimental Tests and Results

In this experiment, the lower back of the human body (about the vertical line of the center of gravity of the human body) is selected as the wearing part of the swimming posture measurement device. After the waterproof protection is completed, it is fixed on the subject with a customized belt. In order to obtain a standard swimming posture signal, the tester chose to be a professional swimming coach, and used different swimming postures to conduct experimental tests in the university swimming pool (the length of the swimming pool is 50 meters).

**Figure 5:**
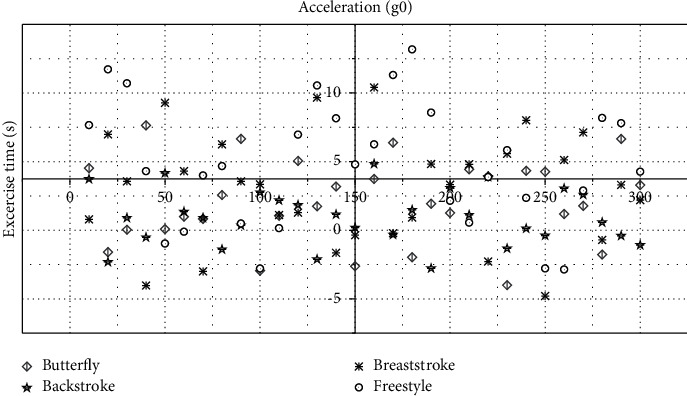
Acceleration signals corresponding to butterfly, backstroke, breaststroke, and freestyle.

**Figure 6:**
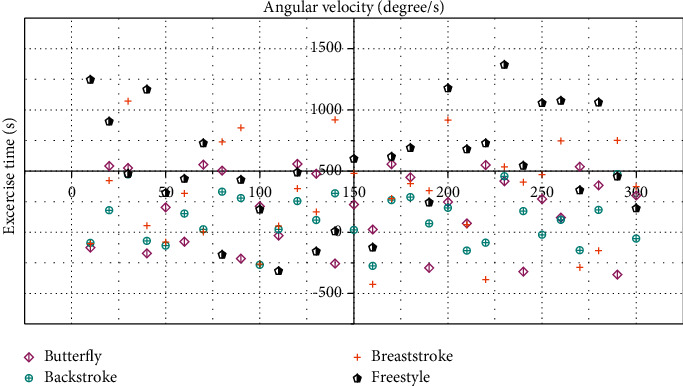
Angular velocity signals corresponding to butterfly, backstroke, breaststroke, and freestyle.

The testers in the experiment used four strokes of high-intensity butterfly, backstroke, breaststroke, and freestyle to test. The measurement device continuously monitors the acceleration signal and gyroscope signal of the tester's body, as shown in Figures 5 and 6.

The swimming posture measurement device in this paper can acquire the three-axis acceleration information and three-axis gyroscope information of the human body in real time when swimming and can provide the rehabilitation therapist with accurate human body condition information after simple analysis. Among them, the acceleration of each axis of the human body does not exceed 15*G*_0_, the angular velocity of each axis does not exceed 1500°/s, and the frequency *f* of the swimming action is below 2 Hz.

### 4.3. Data Analysis

As the *X*-axis of the sensor in the swimming posture measurement device worn is aligned with the central axis of the human body, the *X*-axis data represent the motion information in the forward direction of the human body, the *Y*-axis data represent the motion information in the left and right directions of the human body, and the *Z*-axis data represent the up and down directions of the human body.

According to the motion information of the four strokes, only the *Z*-axis acceleration data corresponding to the backstroke are negative, which is exactly in line with the swimming posture with the face up only in the backstroke; only the *Y*-axis gyroscope data corresponding to the backstroke and freestyle have a large amplitude, which is consistent with the fact that backstroke and freestyle need to turn the body left and right; only the *X*-axis gyroscope data and *Z*-axis acceleration data corresponding to butterfly stroke and breaststroke have large amplitudes, which is consistent with the characteristics that butterfly and breaststroke mainly rely on XZ plane motion. However, the waveform shapes of the *X*-axis gyroscope data corresponding to butterfly and breaststroke are significantly different, which is a result of the different body movements of the two strokes. Therefore, the swimming posture recognition of the human body can be carried out directly according to the aforementioned characteristics of motion information.

**Figure 7:**
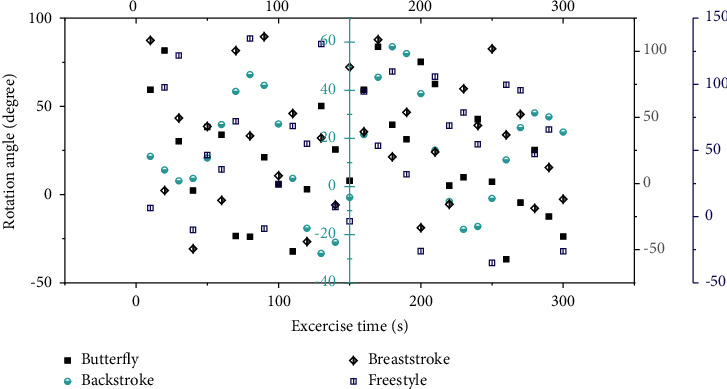
Rotation angle signals corresponding to butterfly, backstroke, breaststroke, and freestyle.

The *X*-axis acceleration amplitude and angular velocity frequency for both strokes increased with increasing swimming intensity. According to the characteristics of backstroke and freestyle strokes, the movements on the left and right sides of the human body are similar and occur alternately; at the same time, the *X*-axis direction of the measuring device is consistent with the central axis of the human body, and the motion information of the *Y*-axis (especially the *Y*-axis angular velocity information) is a strong one. In this paper, the rotation angle data of different strengths and strokes in the experiment are extracted and analyzed as the basic data.  Figure 7 shows the rotation angle signal of the human body relative to the central axis during moderate-intensity backstroke and freestyle.

**Figure 8:**
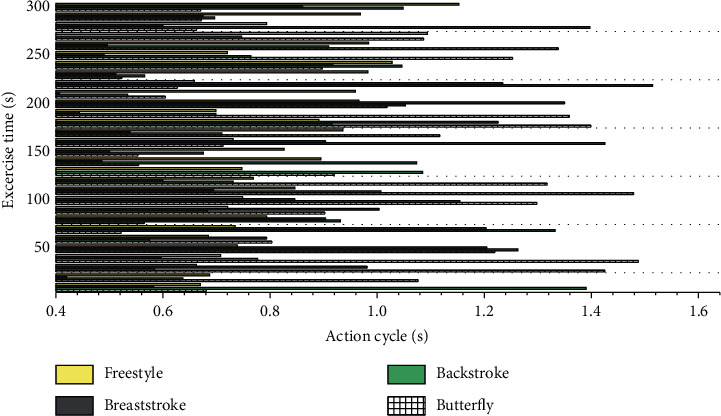
Action cycle diagram corresponding to butterfly, backstroke, breaststroke, and freestyle.

The rotation angle can clearly record the swimming rhythm of the human body and has good signal characteristics. Combined with the signal analysis technology, the signal period, maximum value, and minimum value can also be extracted to represent the swimming action period and the rotation angle to the left and right sides. This information is difficult to directly observe accurately through the human eye and can be provided as feedback information to rehabilitation therapists for swimming rehabilitation training or to professional coaches to prevent daily training injuries of professional athletes. The left-hand motion period TL (the duration of the left-hand motion) and the right-hand motion period TR (the duration of the right-hand motion) are determined according to the time when the rotation angle signal crosses the zero point. In the same way, the human body rotation angle signals in the low-, medium-, and high-intensity backstroke and freestyle exercise information are extracted, respectively, and the maximum value, the minimum value, and the left and right hand action cycles are obtained.  Figure 8 shows the action cycle diagrams corresponding to butterfly, backstroke, breaststroke, and freestyle.

**Figure 9:**
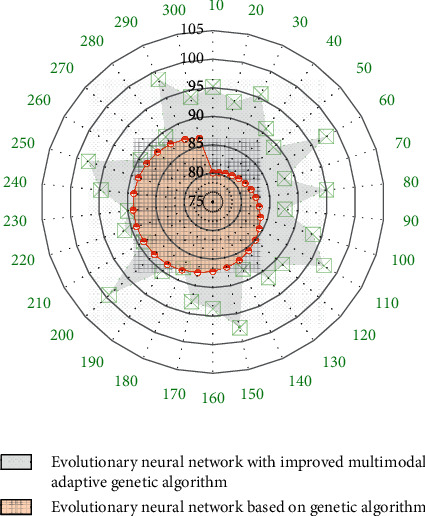
The accuracy of the prediction of the action rotation angle by the evolutionary neural network of the improved multimodal adaptive genetic algorithm.

In swimming rehabilitation training, therapists usually require swimmers to swim at a certain intensity and frequency; in the daily training and movement assessment of professional athletes, coaches also require athletes to complete a series of swimming movements at a certain intensity and frequency. Therefore, in the process of swimming with a specific intensity and frequency, the rotation angle and the action period of the left and right hand movements of the swimmer basically maintain a stable value. If the degree of fatigue of the swimmer increases during swimming, the swimmer will try his best to maintain the original state of motion, but there will be a greater deviation in the accuracy of motion control, so the variance of the rotation angle within a certain period of time can be used to calculate swimmers' fatigue levels. If the swimmer's body has certain spinal diseases, injuries, or limb injuries, there will be a certain degree of asymmetry in the left and right hand movements during swimming. Therefore, the difference between the rotation angle of the left and right hand movements and the difference between the left and right hand movement cycles can be used to evaluate. The accuracy of the evolutionary neural network of the improved multimodal adaptive genetic algorithm on the prediction of the action rotation angle is shown in Figure 9.

## 5. Conclusion

The method of multiphysical domain multimodal information fusion is studied; the problem of multiphysical domain information multimodal fusion is analyzed and described in detail; and the mechanism, structure, process, and mathematical model of multiphysical domain information multimodal fusion are given. The commonly used information fusion methods are compared, and an information fusion method based on the mixture of evolutionary algorithm and neural network is proposed. Aiming at the shortcomings of the neural network, an optimized genetic algorithm combined with a multimodal adaptive evolutionary neural network information fusion method of the neural network is constructed, and the network is used to solve the XOR problem, which proves the effectiveness of the method. In this paper, the carrier motion and attitude detection based on wireless transmission is studied. The efficient data processing function and wide application of MPU6050 make it the basic module for building attitude-detection system. The attitude information of the movement of the carrier is obtained through a plurality of attitude measurement devices installed on the carrier and transmitted after certain data processing. However, the accuracy of data processing in the system needs to be further improved. As the original acquisition itself is limited, it will mainly be upgraded and optimized from the aspects of calculation and processing. In addition, the research on information fusion is still insufficient, failing to process more other modal information. Moreover, handing over the mixed and complex information to the neural network model for direct processing will lose a lot of important information, and data mining can be performed before inputting the model.

## Data Availability

The data used to support the findings of this study are available from the corresponding author upon request.

## References

[1] Xie F., Zhong Y., Du R., Li Z. (2019). Central pattern generator (CPG) control of a biomimetic robot fish for multimodal swimming. *Journal of Bionics Engineering*.

[2] Lienhart R., Einfalt M., Zecha D. (2018). Mining automatically estimated poses from video recordings of top athletes. *International Journal of Computer Science in Sport*.

[3] Pastromas S. (2021). Long QT syndrome in athletes: challenges in the diagnosis and management: long QT syndrome in athletes[J]. *Rhythmos*.

[4] Paganini M., Moon R. E., Boccalon N. (2022). Blood gas analyses in hyperbaric and underwater environments: a systematic review. *Journal of Applied Physiology*.

[5] Dong Z., Bao T., Zheng M., Yang X., Song L., Mao Y. (2019). Heading control of unmanned marine vehicles based on an improved robust adaptive fuzzy neural network control algorithm. *IEEE Access*.

[6] Cronin N. J., Rantalainen T., Ahtiainen J. P., Hynynen E., Waller B. (2019). Markerless 2D kinematic analysis of underwater running: a deep learning approach. *Journal of Biomechanics*.

[7] Wang W., Dai X., Li L. (2018). Three-dimensional modeling of a fin-actuated robotic fish with multimodal swimming. *IEEE*.

[8] Lohan A. (2021). Athletes’ performance with yoga and associated exercises. *Academicia: An International Multidisciplinary Research Journal*.

[9] Lauer J., Vilas-Boas J. P., Rouard A. H. (2018). Shoulder joint kinetics and dynamics during underwater forward arm elevation. *Journal of Biomechanics*.

[10] Godoy L. D., Umeoka E. H. L., Ribeiro D. E. (2018). Multimodal early-life stress induces biological changes associated to psychopathologies. *Hormones and Behavior*.

[11] Dos Santos M. A. M., Henrique R. S., Salvina M. (2021). The influence of anthropometric variables, body composition, propulsive force and maturation on 50m freestyle swimming performance in junior swimmers: an allometric approach. *Journal of Sports Sciences*.

[12] Wang M., Dong H., Li X., Zhang Y., Yu J. (2019). Control and optimization of a bionic robotic fish through a combination of CPG model and PSO. *Neurocomputing*.

[13] Magaia N., Ribeiro I. d. L., de Aguiar A. W. O., Fonseca R., Muhammad K., de Albuquerque V. H. C. (2021). An artificial intelligence application for drone-assisted 5G remote e-health. *IEEE Internet of Things Magazine*.

[14] Liu J., Wu X., Huang C. (2020). 3-d autonomous manipulation system of helical microswimmers with online compensation update[J]. *IEEE Transactions on Automation Science and Engineering*.

[15] Hogarth L., Nicholson V., Payton C., Burkett B. (2021). Modelling the age‐related trajectory of performance in Para swimmers with physical, vision and intellectual impairment. *Scandinavian Journal of Medicine & Science in Sports*.

[16] Sun Y., Zhang X., Xin Q., Huang J. (2018). Developing a multi-filter convolutional neural network for semantic segmentation using high-resolution aerial imagery and LiDAR data. *ISPRS Journal of Photogrammetry and Remote Sensing*.

[17] Zhao T., Zhao J., Zhou W., Zhou Y., Li H. (2021). State representation learning with adjacent state consistency loss for deep reinforcement learning. *IEEE MultiMedia*.

[18] Malawski F., Kwolek B. (2019). Improving multimodal action representation with joint motion history context. *Journal of Visual Communication and Image Representation*.

[19] Zhang P., Wu Z., Dong H., Tan M., Yu J. (2020). Reaction-wheel-based roll stabilization for a robotic fish using neural network sliding mode control. *IEEE*.

[20] Liu J., Xu T., Yang S. X., Wu X. (2019). Navigation and visual feedback control for magnetically driven helical miniature swimmers[J]. *IEEE Transactions on Industrial Informatics*.

[21] Song H., xiu-ying Han C. E., Montenegro-Marin C. E., krishnamoorthy S. (2021). Secure prediction and assessment of sports injuries using deep learning based convolutional neural network. *Journal of Ambient Intelligence and Humanized Computing*.

[22] Wu X., Liu J., Huang C., Su M., Xu T. (2019). 3-D path following of helical microswimmers with an adaptive orientation compensation model[J]. *IEEE Transactions on Automation Science and Engineering*.

[23] Mongeau J.-M., Schweikert L. E., Davis A. L., Reichert M. S., Kanwal J. K. (2021). Multimodal integration across spatiotemporal scales to guide invertebrate locomotion. *Integrative and Comparative Biology*.

[24] Vathagavorakul R., Gonjo T., Homma M. (2021). Differences in limb coordination in polyrhythmic production among water polo players, artistic swimmers and drummers. *Journal of Motor Behavior*.

